# Cross-cultural validity of the generalized anxiety disorder 7-item scale (GAD-7): a systematic review and standardized assessment

**DOI:** 10.1017/S2045796026100560

**Published:** 2026-04-13

**Authors:** Anastasia Mantzari, Lorena Botella-Juan, Prateek Yadav, Grace Lavelle, Ana Henriques, Daniel Munblit, Amy Ronaldson, Alex Dregan, Ioannis Bakolis, Mariana Pinto da Costa, Maria Carmen Viana, Alba Marcos-Delgado, Antonio J Molina, Vicente Martín, Jose M Valderas, Gemma Vilagut, Jordi Alonso, Jorge Arias de la Torre

**Affiliations:** 1 Institute of Psychiatry, Psychology and Neurosciences IoPPN, Kings College London, London, UK; 2 Instituto de Biomedicina IBIOMED, Universidad de Leon, Leon, Spain; 3 CIBER Epidemiologia y Salud Publica CIBERESP ISCIII, Madrid, Spain; 4 South London and Maudsley NHS Foundation Trust, London, UK; 5 EPIUnit ITR, Instituto de Saude Publica, Universidade do Porto, Porto, Portugal; 6 Care in Long Term Conditions Research Division, Kings College London, London, UK; 7 Department of Paediatrics and Paediatric Infectious Diseases, Institute of Childs Health, Sechenov First Moscow State Medical University Sechenov University, Moscow, Russia; 8 Research and Clinical Center for Neuropsychiatry, Moscow, Russia; 9 Department of Collective Health, Federal University of Espirito Santo, Vitoria, Brazil; 10 Department of Medicine, National University of Singapore, Singapore; 11 Department of Family Medicine, National University Health System, Singapore; 12 Centre for Research in Health Systems Performance CRiHSP National University Health System, Singapore; 13 Epidemiology and Public Health, Hospital del Mar Research Institute, Barcelona, Spain; 14 Department of Medicine and Life Sciences, Pompeu Fabra University UPF, Barcelona, Spain

**Keywords:** anxiety, cross-cultural validity, GAD-7, generalized anxiety disorder, systematic review

## Abstract

**Aims:**

The Generalized Anxiety Disorder 7-Item Scale (GAD-7) is a brief self-reported measure for screening for anxiety symptoms. However, the evidence about its cross-cultural validity is fragmentary and usually focused on specific settings. Therefore, we aimed to critically review and synthesize the existing evidence about the cross-cultural validity of the GAD-7.

**Methods:**

We conducted a systematic review of studies assessing the cross-cultural validity of the GAD-7 in following the PRISMA guidelines. Additionally, the quality of the studies was assessed following the COSMIN guidelines, and the quality of the evidence was assessed with the GRADE. Data were synthesized narratively.

**Results:**

Out of 1,965 unique records, 9 unique studies were deemed eligible for the COSMIN appraisal and the narrative synthesis (total sample: 11,894, 53.7% females and 20 different cultural groups). Most studies (7) had adequate quality and showed evidenced of the unitary structure of the GAD-7 across cultural groups. In 4 studies also assessing possible cultural bias, the effect on the general score was deemed negligible.

**Conclusions:**

The evidence about the cross-cultural validity of the GAD-7 is very limited. Although more research is needed, the evidence available shows that the GAD-7 could be a cross-culturally valid tool for the assessment of anxiety symptoms in clinical contexts and epidemiological studies. Until new high-quality evidence will be available, these results would constitute a key first step for supporting the use of the GAD-7 in multi-cultural clinical settings and to inform clinical, public health and global health decision making in relation to anxiety.

## Introduction

Generalized anxiety disorder is one of the most prevalent mental disorders worldwide and represents one of the main causes of morbidity and mortality worldwide (Stein and Sareen, 2015). This disorder has an estimated lifetime prevalence around 3.7% (Ruscio *et al.*, 2017) with a wide variability according to the specific population and population group assessed, and with higher prevalence found in countries with higher economic development (Ruscio *et al.*, 2017).

The measure most widely used for both the screening of generalized anxiety disorder and general anxiety symptoms in the adult population is the Generalized Anxiety Disorder 7-Item Scale (GAD-7; Spitzer *et al.*, 2006; Gregory K. Farber *et al.*, 2023). This self-reported measure consists of seven Likert-type items with a response scale ranging from 0 (*‘Not at all’*) to 3 (*‘Nearly every day’*), originally developed for primary care based on symptoms of generalized anxiety disorder included in the 4th Edition of the Diagnostic and Statistical manual of Mental Disorders (DSM-IV). Additionally, the GAD-7 has been also used in different contexts to screen for anxiety symptoms that can be associated with other mental disorders, such as post-traumatic stress disorder (Kroenke *et al.*, 2007; Plummer *et al.*, 2016) making the GAD-7 a flexible measure for a variety of study designs and settings (Löwe *et al.*, 2008; Ruiz *et al.*, 2011; Johnson *et al.*, 2019; Gregory K. Farber *et al.*, 2023). It is therefore not surprising that the GAD-7 has been prioritized as the standard measure for assessing anxiety by the Common Measures in Mental Health Science (CMMHS) joint initiative developed by funding bodies and scientific journals (Gregory K. Farber *et al.*, 2023).

Originally developed and validated on a predominantly Caucasian (80%) sample of patients from primary care centres across 12 states in the United States (US) (Spitzer *et al.*, 2006) the GAD-7 has been translated and validated in many different languages such as Arabic (Sawaya *et al.*, 2016), Chinese (Zeng *et al.*, 2013), Portuguese (Sousa *et al.*, 2015), Urdu (Ahmad *et al.*, 2017) or Spanish (García-Campayo *et al.*, 2010). It has shown an acceptable validity across different population groups within countries, including Brazil, Germany, Canada, South Africa or India (Löwe *et al.*, 2008; Doi *et al.*, 2018; Henn and Morgan, 2019; De Man *et al.*, 2021; Romano *et al.*, 2022). However, this evidence remains fragmentary as in most of the cases it is derived from studies just focusing on a single country. Additionally, despite the prioritization of the use of the GAD-7 for the screening of anxiety and its extended use worldwide, the evidence on the cross-cultural validity (i.e., the degree to which a questionnaire equivalently and accurately measures the same construct across different cultures or cultural groups) of the GAD-7 remains limited. Thus, a synthesis of the existing but scattered evidence on the cross-cultural comparability of the GAD-7 is essential for providing a more robust evidence for its global use as a screening tool for generalized anxiety disorder and, in general, for anxiety as proposed by CMMHS Initiative (Gregory K. Farber *et al.*, 2023). Additionally, this evidence would provide easy-to-access information on the value of the GAD-7 as an universal tool for the screenings for anxiety in clinical contexts across different cultural backgrounds.

Our aim was to systematically review, synthesize and assess the available evidence about the cross-cultural validity of the GAD-7, to arrive at an evidence-based recommendation about its cross-cultural use for screening.

## Method

### Search strategy and study selection

A systematic review of the literature on the cross-cultural validity of the GAD-7 was carried out following the Preferred Reporting Items for Systematic Reviews and Meta-Analyses guidelines (PRISMA; Page *et al.*, 2021) the COnsensus-based Standards for the selection of health Measurement INstruments (COSMIN) framework (Mokkink *et al.*, 2006) and the Popay *et al.* (2006) guidance on the conduct of narrative synthesis for systematic reviews (Popay *et al.*, 2006). The protocol of the systematic review was registered in the PROSPERO database (registration number: CRD42024536741).

The search was performed on April 21, 2024 in four databases MEDLINE, PubMed, APA PsycINFO and Embase, with no restriction on publication date. Studies assessing the cross-cultural validity of the GAD-7 from a quantitative perspective using primary or secondary data were included. Studies focusing on the assessment of other metric properties, such as reliability or other types of validity, or including study samples from just one culture or one population group, i.e., that did not include a comparison group, were excluded. The search terms used for this review were gathered upon consulting the COSMIN strategy for searching databases for studies of psychometric properties (Terwee *et al.*, 2009; Prinsen *et al.*, 2018) and were linked to two themes: instrument search (terms related to the GAD-7) and outcome search (terms related to cross-cultural validity). Other suggested themes, such as population (general population) and construct (generalized anxiety disorder), were disregarded upon testing as they seemingly generated a high number of irrelevant results. MeSH terms and keywords (i.e., ‘Cross-cultural comparison’, ‘cultural bias’, ‘general anxiety disorder’, ‘GAD-7’) linked by Boolean operators were used for the search. The complete search strategies used in each database are available in Appendix A. The searches were performed by a single researcher (AM). Studies assessing the measurement properties of the GAD-7 or its items and considering possible differences between two or more cultural groups were included. Non-peer reviewed publications and conference abstracts were excluded.

After removing the duplicated records, the eligibility of the studies was established in three consecutive stages. Firstly, all retrieved records underwent a title and abstract review by two independent researchers (AM, LBJ), and the discrepancies were assessed by a third researcher (JAT). Next, a full text review for those documents meeting the inclusion criteria was performed by AM, PY, LBJ. Discrepancies about the selection of a document for the narrative synthesis after the full text reading were resolved by another researcher (JAT) after his evaluation and consultation with other authors. In addition to the systematic search, a manual search by tracking backwards the citations included in the reference list of eligible papers was conducted to try to find potentially relevant documents fitting in the study objective and inclusion and exclusion criteria. Due to the very specific focus of the review the narrative synthesis was focused on summarizing the results of the studies finally selected based on their quality, rather than on identifying and discussing emerging themes different from the cross-cultural validity of the GAD-7.

### Quality assessment

The assessment of the methodological quality of eligible studies was performed by 2 independent reviewers (AM and JAT) using the relevant criteria specific for cross-cultural validity/measurement invariance COSMIN Risk of Bias checklist (Mokkink *et al.*, 2018; Prinsen *et al.*, 2018). These criteria included: (1) whether the group subsamples shared most characteristics apart from the grouping variable (i.e., gender, age, socioeconomic status, potential diagnosis), (2) whether an appropriate methodological approach was deployed to analyse the data and (3) whether the group subsamples were large enough. Each of the studies was rated on each of the three proposed criteria using a four-point scale (very good, adequate, doubtful, inadequate). To determine the overall rating of the methodological quality of each study, the COSMIN manual suggests adopting the ‘worst score counts’ principle, meaning that if a study scored ‘inadequate’ in any criteria, its overall rating should subsequently be considered ‘inadequate’ (Prinsen *et al.*, 2018). This information is depicted in more detail in Appendix B.

In addition to rating the methodological quality, the quality criteria for suitable measurement properties were also examined in the studies finally included in the review, based on COSMIN criteria (Prinsen *et al.*, 2018). Each study was rated as providing sufficient (+), insufficient (–), or indeterminate (?) evidence, based on the analyses conducted and their results in relation to the cross-cultural validity of the GAD-7. The specific quality criteria for consider a questionnaire as a tool with suitable measurement properties are shown in the Appendix C. Upon rating the methodological quality and the outcome for each of the studies finally included based on the study design and statistical methods, the COSMIN manual suggests qualitatively summarizing the evidence and repeating the process for obtaining an overall outcome for the measure (Prinsen *et al.*, 2018). Specifically, the quality criteria for suitable measurement properties were applied to the general summary outcome, indicating whether the GAD-7 has sufficient (+), insufficient (–), inconsistent (±) or indeterminate (?) cross-cultural validity (Prinsen *et al.*, 2018). This rating was based on the ‘75% rule’, meaning that at least 75% of the studies included needed to have found cross-culturally valid results for the GAD-7’s cross-cultural validity to be rated as ‘sufficient’ (Prinsen *et al.*, 2018).

Finally, the evidence leading to the summary outcome was graded as of ‘high’, ‘moderate’, ‘low’, or ‘very low’ quality following a modified version of the Grading of Recommendations, Assessment, Development, and Evaluations (GRADE) approach. (Prinsen *et al.*, 2018). For applying the GRADE approach to the summary result of a measurement property review, the following factors were considered: risk of bias (i.e., the methodological quality of the studies), inconsistency (i.e., unexplained discrepancies across the results of the included studies), imprecision (i.e., potentially low total sample size of the included studies) and indirectness (i.e., evidence deriving from different populations than the population of interest). More detailed information about the COSMIN quality of evidence grading and the GRADE approach can be found in the Appendix D.

### Data extraction and synthesis

Following Popay *et al.* (2006) guidance (Popay *et al.*, 2006) three distinct steps were completed for synthesizing the evidence from the studies finally included. First, a preliminary extraction and synthesis of the results was put together by one author (AM) using tabulation along with textual descriptions and the common rubrics suggested by the COSMIN guidelines for rating the methodological quality of the included studies and the nature of the outcomes. Data extraction included author year, GAD-7 language, study population and sample size, methodology, and main study findings. Additionally, specific psychometric analyses were considered (i.e., Confirmatory Factor Analysis or CFA, Differential Item Functioning or DIF). For the data extraction, floor and ceiling effects, as well as any limitations noted in each paper, were also considered. Second, the potential relationships among the available data were explored through subgroup and thematic analysis, including some further tabulation for highlighting overlapping outcomes across the included studies by two authors (AM, JAT). Third, an assessment of the robustness of the synthesis was conducted (AM and JAT) by applying the GRADE quality rating on the summary outcome and following the ‘best evidence synthesis’ as recommended by both the COSMIN group (Prinsen *et al.*, 2018) and Popay *et al.* (2006), where high-quality studies can weigh more heavily on the summary outcome compared to low-quality ones, especially in case of an inconsistency requiring resolution.

## Results

**Figure 1.**
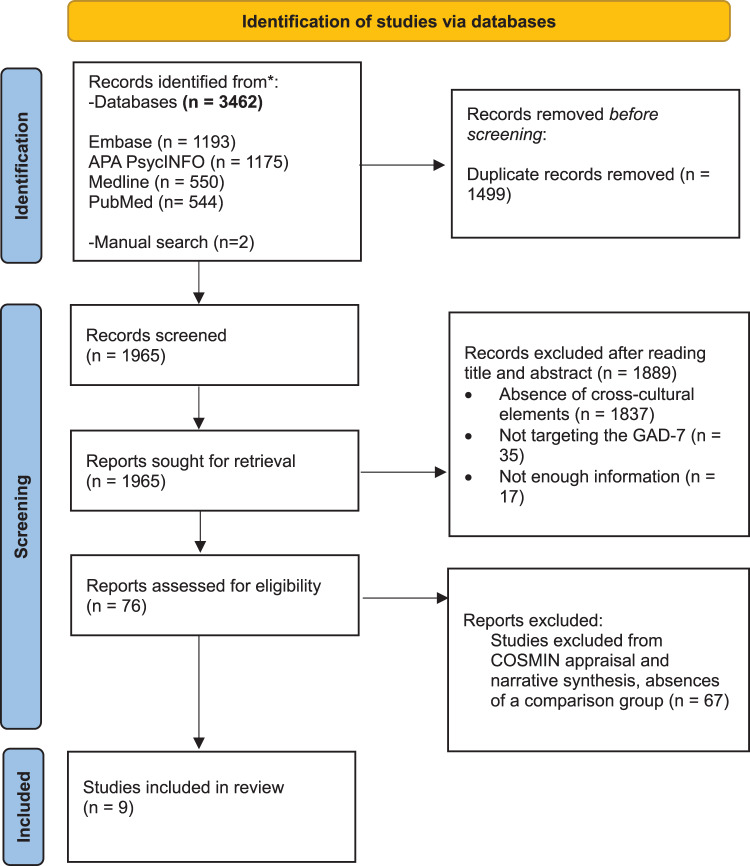
PRISMA flow diagram outlining the screening and selection process for measurement property studies assessing the cross-cultural validity of the GAD-7. Abbreviations: PRISMA: Preferred Reporting Items for Systematic Reviews and Meta-Analyses guidelines; GAD-7: Generalized Anxiety Disorder-7; *n*: number of studies; COSMIN: Consensus-Based Standards for the Selection of Health Measurement Instruments.

The original search yielded 1,965 unique records, of which all underwent title and abstract review while only 76 qualified for the full text review (Fig. 1). The full text review revealed that 67 of these studies lacked a cultural comparison, i.e., that the validity of the GAD-7 was assessed just considering one population group (culture), resulting in 9 included studies effectively assessing the cross-cultural validity of the GAD-7.

The 9 studies analysed included a total sample of 11,894 (53.7% female) individuals (Mills *et al.*, 2014; Parkerson *et al.*, 2015; Henn and Morgan, 2019; Shrestha *et al.*, 2020; Teymoori *et al.*, 2020; Zhou *et al.*, 2020; Sriken *et al.*, 2021; Shevlin *et al.*, 2022; Sahbaz *et al.*, 2024), representing approximately 20 different ethnic groups (e.g., Hispanics/Latins [Mills *et al.*, 2014; Sahbaz *et al.*, 2024] and Black/African americans [Parkerson *et al.*, 2015; Henn and Morgan, 2019; Shrestha *et al.*, 2020]), and languages (i.e., English, Spanish, and French [Teymoori *et al.*, 2020; Shevlin *et al.*, 2022]; Table 1). The primary statistical methods included Single and/or Multi- Confirmatory Factor Analysis (CFA) in 8 studies, and logistic regression for Differential Item Functioning (DIF) in 4 studies. The quality of seven of the studies was deemed ‘adequate’ (the comprehensive COSMIN rating results can be found in the Appendix E; Mills *et al.*, 2014; Parkerson *et al.*, 2015; Teymoori *et al.*, 2020; Zhou *et al.*, 2020; Sriken *et al.*, 2021; Shevlin *et al.*, 2022; Sahbaz *et al.*, 2024).

**Table 1.** Summary of findings and COSMIN appraisal ratings for each of the included studies

					COSMIN appraisal – cross-cultural validity/measurement invariance	
Author (year)	GAD-7 language(s)	Study population and sample size	Methodology	Study findings	Methodological Quality	Outcomes Rating
Mills *et al.* (2014)	English (US) and Spanish	210 Hispanic Americans with English language preference	Multi-group CFA	–The unidimensional factor structure of the GAD-7 provided a good fit for both linguistic groups.	Adequate	Sufficient (+)
		Comparator: 226 Hispanic Americans with Spanish language preference		–Configural, metric, and scalar invariance were examined and evidenced. The factor variance invariance (structural invariance) was also examined and revealed that Hispanic Americans with a Spanish language preference scored higher on the GAD-7 with greater variability. However, these differences disappeared upon controlling for differences in SES between the groups.		
Parkerson *et al.* (2015)	English (US)	673 White/Caucasian young adults (in 2 independent samples) Comparators: A) 174 Hispanic young adults and B) 103 Black/African American young adults	Single-group CFA, DIF (Item characteristics curves between groups)	–Single-group CFAs of the unidimensional factor structure of the GAD-7 provided a good fit for the White/Caucasian and Hispanic groups. Kertz and colleagues’ (2013) revised unidimensional model, which allows for covariance among error terms on somatic-related items (items 4–6), provided a statistically significantly better fit for the Black/African American group. –Item characteristic curves exhibited potentially problematic DIF in items 1, 5, and 6, given that Black/African American individuals with high levels of the latent trait appeared more likely to score lower on these items compared to other participants with similar GAD symptoms. Overall, this would lead Black/African American participants with high GAD levels to a total score approximately 2–3 points lower than other participants.	Adequate	Insufficient (-)
Henn and Morgan (2019)	English (South Africa)	225 White workers Comparator: 304 African workers	DIF (Specific nested logistic regression models for each item)	–Item characteristic curves showed that White workers were more likely than African workers to endorse item 1 and to not endorse item 3. However, the size of these DIF effects were quite small with respect to the change in R^2^ (Δ*R*^2^ < 0.035), suggesting little to no impact at the item level. Item 1 appeared to be especially problematic at the upper end of the distribution	Very Good	Insufficient (−)
Shrestha *et al.* (2020)	English (US)	33 Caucasian older adults	CFA, Independent samples *t-*test	–The unidimensional factor structure of the GAD-7 was confirmed for both racial groups of older adults.	Doubtful	Sufficient (+)
		Comparator: 148 African American older adults		–Independent samples *t*–tests revealed no significant differences in mean scores between African American and Caucasian participants.		
Teymoori *et al.* (2020)	English (UK), Dutch, Finish, Norwegian, German, Spanish, Italian, and French	8 linguistic groups of patients (16+ years) with mild Traumatic Brain Injury (TBI): 207 English, 587 Dutch, 207 Finish, 260 Norwegian, 81 German, 269 Italian, 254 Spanish, and 117 French TBI patients	CFA, DIF [multiple approaches: IRT, Logistic Regression, and the Mantel-Haenszel methods]	–The unidimensional factor structure of the GAD-7 was confirmed across the linguistic groups of traumatic brain injury (TBI) patients.	Adequate	Sufficient (+)
				Teymoori *et al.* (2020) flagged items 3 and 5 for DIF in two of their four DIF tests, but effect sizes were deemed negligible.		
		DIF was performed only to the groups of >200 participants, meaning the English, Dutch, Finish, Norwegian, Italian, and Spanish linguistic groups.				
Zhou *et al.* (2020)	German and Chinese	416 Western (represented by German) university students	Single and Multi-group CFA	–The original unidimensional factor structure of the GAD-7 was confirmed. However, the revised unitary model, which allows for covariance among error terms on somatic-related items (items 4–6), provided a statistically significantly better fit for both groups.	Adequate	Sufficient (+)
		Comparator: 413 Chinese university students		–Configural invariance was examined and evidenced. Metric and scalar invariance were examined and revealed that German students were more likely to score high on items 1, 2, and 4. However, the latent means of the two groups did not differ, meaning that these differences were negligible and that German students did not have significantly higher levels of general anxiety than Chinese students.		
Sriken *et al.* (2021)	English (US)	241 White American university students Comparator: 173 Non-white American university students, including 81 Asian, 52 African American, 22 Hispanic/Latinx, 7 Alaskan Native, 5 Middle Eastern, 3 Pacific Islander, and 3 other. Multi-group CFA was performed only across the two main groups; White and Non-white university students.	Single and Multi-group CFA	–Single-group CFAs indicated a unidimensional factor structure of the GAD-7 for both racial groups. Configural, metric, and scalar invariance were all evidenced across the White and Non-white university students, concluding strong factorial invariance –The Asian, African and Latin descent sample were too small (<100) to perform disaggregated analysis.	Adequate	Sufficient (+)
Shevlin *et al.* (2022)	English (UK), Spanish, and Italian	2,025 UK adults Comparators: A) 1,041 Irish adults, B) 1,949 Spanish adults, and C) 1,039 Italian adults	Single and Multi-group CFA, DIF (MIMIC approach)	–Single-group CFAs confirmed the unidimensional factor structure of the GAD-7 for all four countries. Additionally, configural and metric invariance were examined and evidenced.	Adequate	Sufficient (+)
				–Potential DIF was spotted on item 2, with Spanish participants appearing more likely to score higher than the reference group (UK). However, the size of this effect was rather small and unlikely to contribute to problematic DIF.		
Sahbaz *et al.* (2024)	Spanish	305 Venezuelans residing in Colombia	Multigroup CFA, IRT	–The unidimensional factor structure of the GAD-7 provided a good fit for all three Latino migrant groups.	Adequate	Sufficient (+)
		Comparators: 342 Venezuelans residing in in the United States and 286 Mexicans residing in the United States (non-clinical)		–Configural, metric, and scalar invariance were examined and evidenced, with metric invariance providing a slightly poorer fit for Venezuelans in Colombia compared to the two U.S.-based Latino groups.		
				–IRT results demonstrated that all GAD-7 items yielded positive discrimination and difficulty values across the three groups, while item information functions (IFFs) revealed that item 5 (somatic) was the most informative for Venezuelans in Colombia in line with theories concerning cultural differences in physical versus emotional sensations related to anxiety.		
**Summary outcome**	12 languages – translations/adaptations of the GAD-7	Total sample: 11,894 representing approximately 20 distinct ethnic and linguistic groups	Overall outcome rating: **Sufficient** (+) Quality of evidence: **Moderate**			

COSMIN: Consensus-Based Standards for the Selection of Health Measurement Instruments; GAD-7: Generalized Anxiety Disorder-7; CFA: Confirmatory Factor Analysis; DIF: Differential Item Functioning; IRT: Item Response Theory; SES: socioeconomic status.

Considering the overall outcome (Table 1), the GAD-7 had overall sufficient (+) cross-cultural validity. That is, more than 75% (7 out of 9 studies) presented sufficient (+) outcomes to evidence the unitary factor structure of the GAD-7 across the reference and comparator groups, while not detecting any statistically significant DIF in those studies assessing it. The remaining 2 studies demonstrated insufficient (−) cross-cultural validity upon non-negligible indications (Parkerson *et al.*, 2015; Henn and Morgan, 2019), pointing to the presence of measurement biases against the comparator group (Black/African American and Black/South African) associated with the differential function of some of the questionnaire’s items. Additionally, the individual items of the GAD-7 flagged by the reviewed studies as potentially problematic in relation to cross-cultural validity could be found in Table 2. From the 5 studies testing for differences in the specific items using different approaches (Parkerson *et al.*, 2015; Henn and Morgan, 2019; Teymoori *et al.*, 2020; Zhou *et al.*, 2020; Shevlin *et al.*, 2022), 3 of them pointed out Item 1 (‘*feeling nervous, anxious, or on edge*’) as potentially problematic in relation to the cross-cultural validity (Parkerson *et al.*, 2015; Henn and Morgan, 2019; Zhou *et al.*, 2020) being the most reported item. Items 2 (Zhou *et al.*, 2020; Shevlin *et al.*, 2022), 3 (Henn and Morgan, 2019; Teymoori *et al.*, 2020) and 5 (Parkerson *et al.*, 2015; Teymoori *et al.*, 2020) were reported as potentially problematic in 2 studies each.

**Table 2.** GAD-7 items flagged as potentially problematic in relation to cross-cultural validity

	Mills *et al.* (2014) ^a^	Parkerson *et al.* (2015)	Henn and Morgan (2019)	Shrestha *et al.* (2020)^a^	Teymoori *et al.* (2020)	Zhou *et al.* (2020)	Sriken *et al.* (2021)^a^	Shevlin *et al.* (2022)	Sahbaz *et al.* (2024)^a^
Feeling nervous, anxious, or on edge		✓*	✓*			✓			
Not being able to stop or control worrying						✓		✓	
Worrying too much about different things			✓*		✓				
Trouble relaxing						✓			
Being so restless that it is hard to sit still		✓*			✓				
Becoming easily annoyed or irritable		✓*							
Feeling afraid, as if something awful might happen									

GAD-7: Generalized Anxiety Disorder-7.

a Not specifically tested for Differential Item Functioning (DIF).

* Statistical significant differences (*p*-value < 0.05).

Synthesizing the findings and summarizing the outcomes, altogether, the available evidence appears of ‘moderate’ quality upon applying the GRADE criteria (Table 3). From the 9 studies finally included, 7 had an adequate quality, 1 very good quality, and 1 doubtful quality. No quality points were lost for either risk of bias, as the majority of the studies included were of adequate methodological quality, nor for imprecision, as the total sample size was larger than 100. No points were deducted for inconsistency, as no serious contradiction was found among sufficient and insufficient/inconsistent results that was not resolved after implementing the ‘75% rule’. However, one quality point was lost for indirectness, as all included studies were performed in the desired context and population (non-clinical) except for two which recruited clinical populations (Shrestha *et al.*, 2020; Teymoori *et al.*, 2020).

**Table 3.** COSMIN Appraisal Ratings per Included Study. Comprehensive rating per criterion of methodological quality for each of the included studies

	*Design Requirements:* Were the samples similar for relevant characteristics except for the group variable?	*Statistical Methods:* Was an appropriate approach used to analyse the data?	*Statistical Methods:* Was the sample size included in the analysis adequate?	Overall Rating
Mills et al. (2014)	Adequate	Very Good	Very Good	**Adequate**
Parkerson *et al.* (2015)	Very Good	Adequate	Adequate	**Adequate**
Henn and Morgan (2019)	Very Good	Very Good	Very Good	**Very Good**
Shrestha *et al.* (2020)	Doubtful	Adequate	Doubtful	**Doubtful**
Teymoori *et al.* (2020)	Adequate	Very Good	Very Good	**Adequate**
Zhou *et al.* (2020)	Adequate	Very Good	Very Good	**Adequate**
Shevlin et al. (2022)	Adequate	Very Good	Very Good	**Adequate**
Sriken et al. (2021)	Adequate	Adequate	Adequate	**Adequate**
Sahbaz *et al.* (2024)	Adequate	Adequate	Very Good	**Adequate**

COSMIN: COnsensus-based Standards for the selection of health Measurement Instruments.

## Discussion

To our knowledge this is the first study to date that has compiled, assessed and synthesized, the available literature regarding the cross-cultural validity of the GAD-7. Overall, while the evidence about its cross-cultural validity is very limited, the GAD-7 appears to be a cross-culturally valid tool for the assessment of generalized anxiety disorder and anxiety symptoms. The results also highlight a disproportionate paucity of studies on its cross-cultural validity when compared to the vast numbers of studies applying GAD-7 across multiple different cultural contexts. Due to the prioritization of the GAD-7 as a screening measure for anxiety disorders and until new high-quality evidence will be available, these results constitute a key first step for supporting its use for the assessment of anxiety in multi-cultural clinical and community settings, and therefore could constitute the necessary evidence-base for its use in public health and global health decision making in relation to anxiety.

From a public health perspective, the results found could serve to reassure researchers and clinicians that, overall, is likely that the same construct is being measured across different cultures when using the GAD-7. Ensuring the validity of the measure could be helpful to identify and compare the rates of generalized anxiety disorder and anxiety symptoms in different cultural contexts, therefore, aiding the screening and prevention of anxiety disorders at a population level. Besides, considering the cross-cultural performance of the specific items of the GAD-7, the differences found for item 1 between black and white participants in two different contexts could suggest a possible ceiling effect, and this could reflect a lower severity of GAD symptoms than is experienced. As pointed out by previous research (Lewis-Fernández *et al.*, 2010; Marques *et al.*, 2011; Hofmann and Hinton, 2014), these differences could be related to a variety of factors including differing cultural somatic awareness, differing perceptions of stigma or mental health, differing cultural descriptions of anxiety symptoms, or differing use of language. Regarding this, clinicians should take this in count in this specifically populations. However, while further specific research in this area is required, it should be highlighted that the magnitude of the DIF found was small and do not call into question the overall performance of the GAD-7 and its cross-cultural validity as a screening tool for anxiety.

The external validity of the study findings should be discussed as most of the articles reviewed used convenience samples. This convenience sampling allowed the access to large comparable samples and subsamples but compromised the generalizability and representativeness of the findings to other settings and populations. Besides, 6 out of the 9 studies (Parkerson *et al.*, 2015; Henn and Morgan, 2019; Shrestha *et al.*, 2020; Teymoori *et al.*, 2020; Zhou *et al.*, 2020; Sahbaz *et al.*, 2024) were secondary data analyses, i.e., analyses of data collected for other purposes rather than for the study of the cross-cultural validity of the GAD-7. This data reuse could produce their own biases for the study of cross-cultural differences of a measure, particularly in relation to the sample selected and the possible representation and comparability of the different population groups. However, while further representative studies to assess the cross-cultural validity of the GAD-7 are needed, the results derived from convenience samples could serve as a feasible alternative to provisionally support its suitability to be used for the screening of anxiety symptoms in multi-cultural settings.

Different study limitations must be discussed. First, those associated with the COSMIN method used that, for example, has attracted some discussion and criticism around being reliant more on expert opinion than evidence, its focus on classical test theory, and its insufficient focus on underlying constructs measured (McKenna and Heaney, 2021; Mokkink *et al.*, 2021). However, the COSMIN initiative guidelines are one of the most rigorous approaches for assessing the metric properties of a measure, and that these guidelines follow a continuous revision process making them a suitable alternative for this assessment. Another limitation that should be mentioned is that all the studies reviewed were considered equally for estimating the overall outcome, since the majority exhibited adequate methodological quality (Mokkink *et al.*, 2006). For the purposes of the ‘best evidence synthesis’, we initially considered weighing down or excluding one study that receive a COSMIN rating of ‘doubtful’ quality due to its disproportionate reference and comparator groups in terms of size and within group characteristics.(Shrestha *et al.*, 2020). However, due to its valuable sample we decided not weight down the study, making it one of the only reviewed studies to contribute substantial participant data from hard-to-reach communities that often end up underrepresented in research. Besides, the limitation in relation to specific analyses not focused on the assessment of cross-cultural validity, such as content validity analyses, could be mentioned. While these analyses could show critical aspects of any measure, the primary focus of this review was to assess studies that had already implemented adapted versions of the GAD-7, and to analyse their cross-cultural validity. Moreover, the content validity and other relevant metric properties of the GAD-7 have been previously established in its original development and initial adaptations. As such, this review assumes that the included studies have built upon this foundational work. Finally, it is important to acknowledge that although the present review contains studies including 20 distinct cultural groups, only one study was carried out outside the US and Europe (Henn and Morgan, 2019), limiting the generalizability of this review globally and indicating a clear knowledge gap in the literature. Despite this, given the wide range of cultures included and until new high-quality evidence will be available, the results from this review offer a preliminary perspective on the cross-cultural validity of the GAD-7, and could be considered key evidence to support its use from a global perspective.

In conclusion, this review highlights an important evidence gap in relation to the cross-cultural performance of the GAD-7. Additionally, while with limitations, the evidence available to date points out that the GAD-7 is likely to be a cross-culturally valid measure for the screening of generalized anxiety disorder and, in general, of anxiety. This information could be helpful for clinicians, researchers and decision makers to accurately assess, compare and therefore address data on anxiety disorders as a clinical and public health issue using a brief and simple instrument. Therefore, while further primary research in this area is needed to strengthen the evidence base about the cross-cultural validity of the GAD-7, the results from this review could serve to provisionally support its use in multi-cultural settings, and to inform clinical, public health and global mental health decision making in relation to anxiety.

## Data Availability

All the data derived from the study and the materials used for its development are available upon request to the corresponding author.

## **Appendix A.** Search filter and results per database

***Medline***

Search conducted in Medline (via Ovid): 21 April 2024

Results retrieved: 550

Search: ((exp Psychometrics/OR exp Cross-cultural comparison/OR exp Reproducibility of results/OR exp Culture/OR exp Translating/OR exp Factor Analysis, Statistical/OR exp Ethnicity/ OR “Psychometric properties”.mp. OR “Cross-cultural validity”.mp. OR “Cross-cultural validation” .mp. OR “Cross-cultural comparability”.mp. OR “Cross-cultural equivalence”.mp. OR “Cross-cultural measurement invariance”.mp. OR “Cultural difference*”.mp. OR “Cultural bias*”.mp. OR “Construct validity” .mp. OR “Factor structure”.mp. OR “Differential item functioning” .mp. OR DIF.mp. OR “Item response theory” .mp. OR “Confirmatory factor analysis” .mp. OR “Exploratory factor analysis”.mp. OR Translation .mp. OR “Cultural adaptation” .mp. OR Validation .mp. OR “Measurement invariance”.mp.) AND (“Generalized Anxiety Disorder-7”.mp. OR “Generalized Anxiety Disorder-7 (GAD-7)”.mp. OR “General Anxiety Disorder-7”.mp. OR “General Anxiety Disorder-7 (GAD-7)”.mp. OR GAD-7.mp. OR GAD7.mp.))

***PubMed***

Search conducted in PubMed: 21 April 2024

Results retrieved: 544

Search: ((“Psychometrics “[mesh] OR “Cross-cultural comparison “[mesh] OR “Ethnicity”[mesh] OR “Reproducibility of results “[mesh] OR “Culture “[mesh] OR “Translating “[mesh] OR “Factor Analysis, Statistical “[mesh] OR “Validation Study”[mesh] OR “Psychometric properties”[tiab] OR “Cross-cultural validity”[tiab] OR “Cross-cultural validation “[tiab] OR “Cross-cultural comparability “[tiab] OR “Cross-cultural equivalence”[tiab] OR “Cross-cultural measurement invariance”[tiab] OR “Cultural difference*”[tiab] OR “Cultural bias*”[tiab] OR “Construct validity “[tiab] OR “Factor structure”[tiab] OR “Differential item functioning “[tiab] OR “DIF”[tiab] OR “Item response theory “[tiab] OR “Confirmatory factor analysis “[tiab] OR “Exploratory factor analysis”[tiab] OR “Cultural adaptation “[tiab] OR “Validation “[tiab] OR “Measurement invariance”[tiab]) AND (“Generalized Anxiety Disorder-7”[tiab] OR “Generalized Anxiety Disorder-7 (GAD-7)”[tiab] OR “General Anxiety Disorder-7”[tiab] OR “General Anxiety Disorder-7 (GAD-7)”[tiab] OR “GAD-7”[tiab] OR “GAD7”[tiab]))

***APA PsycINFO***

Search conducted in APA PsycINFO (via Ovid): 21 April 2024

Results retrieved: 1175

Search: ((exp Psychometrics/OR exp Cross Cultural Validity/ OR exp Cross Cultural Differences/ OR exp Cross Cultural Test Adaptation/OR exp Item Response Theory/OR exp Classical Test Theory/OR exp Differential Item Functioning/OR exp “Item Analysis (Statistical)”/ OR exp Measurement Invariance/OR exp Ethnic Identity/OR exp Foreign Language Translation/OR exp Factor Structure/OR exp Cultural Test Bias/ OR exp Cultural Bias/OR exp “Racial and Ethnic Differences”/OR exp Construct Validity/OR exp Test Bias/OR exp Confirmatory Factor Analysis/OR exp Exploratory Factor Analysis/ OR exp Adaptation/OR Psychometrics.mp. OR “Psychometric properties”.mp. OR “Cross-cultural validity”.mp. OR “Cross-cultural validation” .mp. OR “Cross-cultural comparability”.mp. OR “Cross-cultural equivalence”.mp. OR “Cross-cultural measurement invariance”.mp. OR “Cultural difference*”.mp. OR “Cultural bias*”.mp. OR “Construct validity” .mp. OR “Factor structure”.mp. OR “Differential item functioning” .mp. OR DIF.mp. OR “Item response theory” .mp. OR Ethnicity .mp. OR Translation .mp. OR Adaptation.mp. OR “Cultural adaptation” .mp. OR Validation .mp. OR “Measurement invariance”.mp.) AND (“Generalized Anxiety Disorder-7”.mp. OR “Generalized Anxiety Disorder-7 (GAD-7)”.mp. OR “General Anxiety Disorder-7”.mp. OR “General Anxiety Disorder-7 (GAD-7)”.mp. OR GAD-7.mp. OR GAD7.mp.))

***Embase***

Search conducted in Embase (via Ovid): 21 April 2024

Results retrieved: 1193

Search: ((exp Psychometry/OR exp Cultural Factor/OR exp Ethnicity/ OR exp “Ethnic of Racial Aspects”/OR exp Ethnic Group/OR exp Ethnic Identity/OR exp Cultural Bias/OR exp Construct Validity/OR exp Factorial Analysis/OR exp Confirmatory Factor Analysis/OR exp Exploratory Factor Analysis/OR exp Validation Study/OR exp Language/OR Psychometrics.mp. OR “Psychometric properties”.mp. OR “Cross-cultural validity”.mp. OR “Cross-cultural validation” .mp. OR “Cross-cultural comparability”.mp. OR “Cross-cultural equivalence”.mp. OR “Cross-cultural measurement invariance”.mp. OR “Cultural difference*”.mp. OR “Cultural bias*”.mp. OR “Factor structure”.mp. OR “Differential item functioning” .mp. OR DIF.mp. OR “Item response theory” .mp. OR Translation .mp. OR “Cultural adaptation” .mp. OR “Measurement invariance”.mp.) AND (“Generalized Anxiety Disorder-7”.mp. OR “Generalized Anxiety Disorder-7 (GAD-7)”.mp. OR “General Anxiety Disorder-7”.mp. OR “General Anxiety Disorder-7 (GAD-7)”.mp. OR GAD-7.mp. OR GAD7.mp.))

## **Appendix B.** COSMIN risk of bias checklist box 5 – cross-cultural validity/measurement invariance

**Table:**

	Very good	Adequate	Doubtful	Inadequate	N/A
*Design Requirements* Were the samples similar for relevant characteristics except for the group variable?	Evidence provided that samples were similar for relevant characteristics except group variable	Stated (but no evidence provided) that samples were similar for relevant characteristics except group variable	Unclear whether samples were similar for relevant characteristics except group variable	Samples were NOT similar for relevant characteristics except group variable	–
*Statistical Methods* Was an appropriate approach used to analyse the data?	A widely recognized or well justified approach was used	Assumable that the approach was appropriate, but not clearly described	Not clear what approach was used or doubtful whether the approach was appropriate	Approach NOT appropriate	–
*Statistical Methods* Was the sample size included in the analysis adequate?	Regression analyses or IRT/Rasch based analyses: 200 subjects per group MGCFA*: 7 times the number of items, and ≥100	150 subjects per group 5 times the number of items, and ≥100; OR 5–7 times the number of items, but <100	100 subjects per group 5 times the number of items, but <100	<100 subjects per group <5 times the number of items	–

COSMIN: COnsensus-based Standards for the selection of health Measurement Instruments; IRT: Item Response Theory; MGCFA: Multigroup Confirmatory Factor Analysis; N/A: Not applicable.

## **Appendix C.** COSMIN criteria for good measurement properties

**Table:**

Measurement property	Rating	Criteria
**Cross-cultural validity/measurement invariance**	**+**	No important differences found between group factors (such as age, gender, language) in multiple group factor analysis OR no important DIF for group factors (McFadden’s R2 < 0.02)
	**?**	No multiple group factor analysis OR DIF analysis performed
	**–**	Important differences between group factors OR DIF was found

COSMIN: COnsensus-based Standards for the selection of health Measurement Instruments; DIF: Differential Item Functioning.

## **Appendix D.** COSMIN quality of evidence grading – the GRADE approach

**Table:**

Quality level	Definition	Lower if
High	We are very confident that the true measurement property lies close to that of the estimate of the measurement property.	Risk of bias −1 Serious −2 Very serious −3 Extremely serious
Moderate	We are moderately confident in the measurement property estimate: the true measurement property is likely to be close to the estimate of the measurement property, but there is a possibility that it is substantially different.	Inconsistency −1 Serious −2 Very serious
Low	Our confidence in the measurement property estimate is limited: the true measurement property may be substantially different from the estimate of the measurement property.	Imprecision −1 total *n* = 50–100 −2 total *n* < 50
Very low	We have very little confidence in the measurement property estimate: the true measurement property is likely to be substantially different from the estimate of the measurement property.	Indirectness −1 Serious −2 Very serious

COSMIN: COnsensus-based Standards for the selection of health Measurement Instruments; GRADE: Grading of Recommendations, Assessment, Development, and Evaluations; *n* = sample size.
